# Characterization of an olfactory system dysfunction model: a vanadium dose-effect study via nose-to-brain delivery in rats

**DOI:** 10.3389/fnana.2025.1641228

**Published:** 2025-09-22

**Authors:** Margarida Pereira, Carlos Venâncio, Maria Lurdes Pinto, Luís Manuel Félix, Sofia Alves-Pimenta, Bruno Colaço

**Affiliations:** ^1^Department of Animal Sciences, School of Agrarian and Veterinary Sciences, University of Trás-os-Montes and Alto Douro (UTAD), Vila Real, Portugal; ^2^Centre for the Research and Technology of Agro-Environmental and Biological Sciences, CITAB, Inov4Agro, University of Trás-os-Montes and Alto Douro (UTAD), Vila Real, Portugal; ^3^CECAV – Animal and Veterinary Research Centre, Associate Laboratory for Animal and Veterinary Sciences (AL4AnimalS), University of Trás-os-Montes and Alto Douro (UTAD), Vila Real, Portugal

**Keywords:** animal model, olfactory dysfunction, nose-to-brain delivery, rat, olfactory system

## Abstract

**Introduction:**

The olfactory system acts as an interface between the environment and the brain. Its direct neural connection makes it a target for xenobiotics and a suitable model for studying olfactory dysfunction and related neurotoxic effects. This study aimed to characterize an animal model of olfactory dysfunction induced by nose-to-brain (NTB) delivery of vanadium pentoxide (V_2_O_5_).

**Methods:**

Rats received 182 or 273 μg intranasally, thrice weekly over 4 weeks, followed by behavioral, histological, and biochemical analysis of the olfactory epithelium (OE), olfactory bulbs (OBs), and hippocampus.

**Results:**

Behavioral tests showed significant olfactory deficits, longer latencies, and reduced investigation times in exposed groups. Histological analysis revealed coagulative necrosis in the OE, disrupted cellular organization, reduced number and size of OB glomeruli, and hippocampal neuronal loss with gliosis. Immunohistochemistry revealed increased proliferating cell nuclear antigen (PCNA) expression in the OE, dopaminergic neuron loss and astroglial proliferation in the OB, and hippocampal astroglial proliferation at the highest dose. Myelin basic protein (MBP) expression remained unchanged. Oxidative stress markers were largely unaltered, except for increased superoxide dismutase (SOD) in OBs and glutathione S-transferase (GST) in the hippocampus, especially at the high dose.

**Discussion:**

The results reveal dose-dependent vanadium-induced neurotoxicity in the olfactory system. The higher dose induced pronounced structural damage, neuroinflammation, and oxidative stress, resulting in olfactory and cognitive impairments relevant to advanced olfactory dysfunction and neurodegeneration. The lower dose induced milder yet significant effects, supporting its use in early-stage dysfunction studies. This NTB-based model offers a valuable tool for investigating olfactory dysfunction mechanisms in toxicological and neurodegenerative contexts.

## 1 Introduction

The olfactory system is the only part of the central nervous system (CNS) directly exposed to the external environment, making it highly susceptible to airborne agents (Doty, 2015). It comprises the olfactory epithelium (OE), which lines the posterior part of the nasal cavity, and specific olfactory brain regions, with the olfactory bulbs (OBs) being the first relay center. Within the OE, olfactory sensory neurons (OSNs) extend their axons through the cribriform plate, forming olfactory nerve bundles that reach the OB. There, these sensory neurons converge to form glomeruli, the initial processing units of olfactory information (Dorman, 2018; Forrester et al., 2018; Lodovichi, 2021). This unique anatomical and physiological organization has positioned the olfactory pathway as a direct interface between the environment and the brain, playing a key role not only in olfaction but also in susceptibility to xenobiotics and CNS-targeted interventions. Given its direct access to the brain, the intranasal route has been explored for both experimental and therapeutic purposes, bypassing the blood-brain barrier and enabling direct nose-to-brain (NTB) transport (Illum, 2012). Through this pathway, substances can reach the brain via intraneuronal transport—where OSNs uptake and transport substances axonally, or through extraneuronal diffusion between neurons (Lochhead and Thorne, 2012; Khan et al., 2017). This direct neural connection that enables NTB transport also serves as a gateway for environmental xenobiotics, which can enter the brain via olfactory receptors or perineural spaces. These compounds have the potential to impair olfactory function, disrupt neurotransmitter activity, and induce neuropathological changes, often resembling those seen in neurodegenerative diseases (Doty, 2015).

Due to its promising potential, NTB delivery has been extensively explored in rodent’s models, demonstrating its capacity to transport various substances to the brain, including small-molecule drugs, peptides, proteins, stem cells, viruses, nucleotides, and numerous toxic substances (Gänger and Schindowski, 2018). In this context, rodent models of NTB have been used for detecting and testing nasal drug absorption and permeation, as well as for pharmacokinetic studies, toxicological and electrophysiological assessments, and evaluation of drug transporter interactions (Erdö et al., 2018). Moreover, neurotoxin models are widely used to investigate the mechanisms underlying olfactory impairment, including disruptions in neurotransmitter systems and neuronal loss in CNS regions involved in olfactory processing (Rey et al., 2018; Li et al., 2019; Prediger et al., 2019; Bhatia-Dey and Heinbockel, 2021). Previous studies have shown that xenobiotics and pathogens can access the CNS through OSNs and propagate trans-synaptically, a mechanism associated with the initiation of neurodegenerative diseases such as Parkinson’s and Alzheimer’s (Colín-Barenque et al., 2015; Rey et al., 2018; Ngwa et al., 2024). This route of entry, combined with the subsequent induction of oxidative stress, neuroinflammation, and neuronal loss, reflects early pathological processes described in these conditions. These findings underscore the need for experimental models that not only evaluate olfactory toxicity but also help elucidate mechanisms potentially involved in neurodegenerative disease onset.

Despite anatomical differences between rodents and humans, the fundamental NTB pathways are similar in both species. This translational relevance underscores the role of animal models in advancing our understanding of NTB delivery, providing essential insights into transport mechanisms, efficacy, and safety before progressing to human applications (Dhuria et al., 2010). However, to fully harness the experimental and therapeutic potential of NTB delivery, several critical aspects must be elucidated. Quantitative studies on efficacy, safety, reproducibility, pharmacokinetics, and drug transport mechanisms are essential to ensure its successful and reliable application in translational research (Ruigrok and de Lange, 2015; Stützle et al., 2015; Flamm et al., 2022). This has motivated the development of refined delivery methods, as traditional approaches using drops or pipettes often lack precision and result in inconsistent targeting of the OE. Such variability limits the accuracy of distribution studies and the interpretation of neurofunctional outcomes. To address these limitations, catheter-based NTB delivery techniques have been developed to enable localized and reproducible substance deposition in the OE, minimizing off-target effects and improving experimental control (Flamm et al., 2018, 2022; Pereira et al., 2024). Nonetheless, further standardization of administration protocols and dose quantification remains necessary.

Given the importance of the olfactory system not only in neurotoxicology but also in neurological disorders, where olfactory dysfunction is recognized as an early marker of neurodegenerative conditions (Goldstein et al., 2010; Ngwa et al., 2014), experimental models that replicate both peripheral and central olfactory impairments are particularly valuable. Our work seeks to address a gap in the literature, as existing NTB delivery models in rodents fail to fully characterize olfactory dysfunction across the entire olfactory pathway. Most models do not account for the complex interactions within the olfactory system, making it difficult to assess the full extent of toxic effects. To overcome this, we selected vanadium (V) as a toxic agent due to its environmental relevance and established neurotoxic properties. Epidemiological studies have linked airborne exposure to olfactory dysfunction, cognitive impairment, and increased neuroinflammatory markers. Experimental findings support its potential to induce oxidative stress, dopaminergic neuron degeneration, and memory deficits in rodent models. Based on this evidence, we administered two different V doses to evaluate their effects and employed a previously refined NTB delivery technique with direct deposition onto the OE in rats, ensuring precise targeting of the olfactory region. To our knowledge, no previous study has utilized this approach to assess the toxicological effects of NTB delivery directly on the olfactory region in rats. In this study, we focus on achieving a robust and detailed characterization of a reproducible model of V-induced olfactory dysfunction. This includes evaluating key structures affected by V toxicity, such as the OE, OBs, and hippocampus. Through this approach, we aim to compare the effects of the two doses to determine whether they induce distinct neurotoxic outcomes and to validate this model and the administration technique for future applications in translational research.

## 2 Materials and methods

### 2.1 Animals

A total of twenty two adult male *Wistar* rats, obtained from the Institute for Research and Innovation in Health, University of Porto, Portugal, were used. The animals were housed and maintained under controlled environmental conditions of temperature (20 ± 2°C), relative air humidity (55 ± 10%), with a 12/12 h light-dark cycle, fed with a standard rodent diet and water *ad libitum*. The experimental procedures were carried out in accordance with the guidelines of the European Directive 2010/63/EU, Portuguese Agency for Animal Welfare (DGAV – n° 013159) and approved by the Ethics Committee of the University of Trás-os-Montes and Alto Douro (ORBEA 10-06-2021). To establish humane endpoints for the experimental protocol and ensure animal welfare, an animal monitoring sheet was developed for daily recording of each animal’s biological parameters. These parameters include body weight, body condition, mental status, coat and grooming, eyes, mucosa, response to handling, respiratory rate, and hydration status, presence of convulsions, body temperature, feces, and urine. Throughout the experiment body weights were measured before the first administration and on the day of each subsequent intranasal administration. The percentage of body weight gain was calculated using the formula (Final body weight-Initial body weight)/Final body weight × 100 (Faustino-Rocha et al., 2013). To determine the food and water intake, the amounts consumed were weighed weekly using a precision lab scale.

### 2.2 Experimental groups and treatments

Solutions of V_2_O_5_ were prepared using powder purchased from Sigma-Aldrich (St. Louis, MO, USA). Rats were randomly assigned to one of three experimental groups: one group (*n* = 7) received an intranasal administration of 273 μg of V_2_O_5_ in 30 μl of distilled water; another group (*n* = 8) received 182 μg of V_2_O_5_; and a control group (*n* = 7) received an equal volume of distilled water. The method of intranasal administration and the instilled volume were adapted from the protocol described by Pereira et al. (2024). The dose of 182 μg V_2_O_5_, as well as the administration frequency and duration, were selected based on previous studies by Ngwa et al. (2014), which reported functional and histological effects in mice. The higher dose of 273 μg V_2_O_5_ was defined based on preliminary trials conducted prior to the experimental protocol to ensure a detectable biological response without causing overt toxicity. Each dose was administered to the left and right nostrils by instillation using a cannula attached to a micropipette, three times a week for 4 weeks. The rats were anesthetized with isoflurane to prevent a gag reflex during the procedure.

### 2.3 Behavioral tests

To evaluate the effects of intranasal V_2_O_5_ administration on the olfactory system, the buried food test and the olfactory habituation/cross-habituation test were conducted to assess the animals’ ability to detect and differentiate various odorants. Additionally, the burrowing test was performed to identify behavioral changes associated with olfactory dysfunction and hippocampal cytotoxic lesions. Two days after the final administration, rats from each group were subjected to each test during the dark phase.

#### 2.3.1 Buried food test

The buried food test was performed to assess the ability of the rats to smell volatile odorants and their tendency to use olfactory cues for foraging (Yang and Crawley, 2009). In the test was used a food pellet with 6 g approximately. The chamber consisted of a clean standard plastic cage (38 × 24 × 20 cm) with a 5 cm layer of new bedding. After 24 h of food deprivation, a rat was placed in the chamber for 10 min to acclimate. Then, the rat was removed from the cage and the food pellet was buried in the bedding, approximately 2 cm beneath the surface, at a random location. The bedding surface was smoothed out and the rat was re-introduced into the cage. The time spent to locate the buried cookie was recorded. The maximum test time allowed was 900 s.

#### 2.3.2 Olfactory habituation/cross-habituation test

This test was performed to assess the rat’s ability to detect and distinguish between a known and a new odor (Yang and Crawley, 2009). Before testing, the rat underwent a 30-min acclimation period to a clean testing plastic cage (38 × 24 × 20 cm), in which was placed through the water bottle opening a plastic tube (8 × 0.5 cm), containing a swab cotton tip inside. The test consisted of sequential presentations of water, two non-social odors and two social odors. Each odor was presented in three consecutive trials of 2 min, with 1-min inter-trial intervals (Wesson et al., 2008; Yang and Crawley, 2009). The sequence used was water, eugenol extract (100 μL, 1:100 dilution in distilled water), almond extract (100 μL, 1:100 dilution in distilled water) and two different social male odors obtained by swabbing a cotton tip on the bed of two different cages with male rats. The total time spent sniffing the odorant during each presentation was measured using a stopwatch during a 2-min interval.

#### 2.3.3 Burrowing test

The burrowing test was performed to measure the innate behavior of the rats and assess the integrity of hippocampal function (Deacon, 2006). The burrowing apparatus consisted of a plastic tube, 320 mm long and 100 mm in diameter, with one open end raised above the cage floor and the other end closed (Deacon, 2006). For acclimatization, prior to testing the effects of intranasal V_2_O_5_ delivery on burrowing behavior, a tube filled with 2000 g of cage floor bedding was placed in the home cage of each group for 3 days. This helped develop burrowing behavior and reduced variability between animals. Testing began at the start of the dark cycle, with a single rat placed in each cage-burrow. After 2 h, the amount of material remaining in the burrow was measured.

### 2.4 Nasal cavity and brain tissue collection

Following behavioral testing, rats were anesthetized with sodium pentobarbital (200 mg/kg) intraperitoneally and then sacrificed by cardiac exsanguination. The brains were removed from the skulls and weighed. Following this, they were separated along the midsagittal plane, resulting in the division of each brain into its right and left hemispheres. The right hemispheres were immersed and fixed in 10% paraformaldehyde for 72 h for histological and immunohistochemical analysis. From the left brain hemisphere, the OB and hippocampus were carefully dissected, frozen individually immediately after collection, and stored at –80 °C for later biochemical analysis. Simultaneously, the rats’ heads were fixed and decalcified at room temperature for 7 days using a commercial fixative and decalcifying solution (OSTEOFAST1, BioGnost^®^, Croatia) to allow histological and immunohistochemical analysis of OE.

### 2.5 Histological and immunohistochemical analysis

For histological and immunohistochemical analysis of the OE, cross-sections of the nasal cavity were prepared at level five, following the standardized protocol described by Popp and Monteiro-Riviere (1985), to ensure consistent anatomical evaluation. For brain analysis, the right hemisphere was coronally sectioned along its rostrocaudal axis, following anatomical guidelines adapted from Rao et al. (2011). The tissue was trimmed to obtain coronal sections corresponding to the olfactory bulbs and hippocampus, as defined in the protocol. This orientation ensured accurate inclusion of both the olfactory bulbs and hippocampal formation in the histological sections. Both fixed nasal cavity and brain cross sections were subsequently dehydrated through a graded ethanol series, cleared in xylene, and embedded in paraffin with the anterior surface oriented downward. Sections were cut at a thickness of 3 μm using a microtome and mounted on glass slides. Histological evaluation of the OE, OBs, and hippocampus was performed on hematoxylin–eosin-stained sections using an optical microscope (Nikon Alphaphot-2 YS2, Japan). In the OE and OBs, the quantification of OSNs, as well as the number and size of glomeruli and mitral cells, was performed through high-magnification direct observation of at least five non-overlapping, randomly selected fields per animal. In the hippocampus, neuronal damage evaluation was performed by high-magnification direct observation of at least seven non-overlapping, randomly selected fields per animal, with attention to neuronal density and the presence of morphological indicators of cell damage, such as pyknotic nuclei and eosinophilic cytoplasm. Lesions in all regions were classified based on the extent of the affected area and cellular pathology, and categorized as none, moderate, or severe.

For immunohistochemistry, paraffin-embedded brain sections were deparaffinized with xylene and rehydrated through a series of graded alcohols. Endogenous peroxidase activity was inhibited by incubating the sections in 3% H_2_O_2_ for 30 min at room temperature. Antigen retrieval was performed by microwaving tissue sections during 5 min in citrate buffer (pH 6 ± 0.2) at 750 Watts. Subsequently, the slides were allowed to cool down at room temperature for 30 min. Slides were washed in PBS and the following steps were performed using the “MACH-1 Universal HRP Polymer Detection Kit” from Biocare^®^, according to the manufacturer’s instructions. Sections were incubated overnight at 4°C with mouse monoclonal antibodies (Santa Cruz Biotechnology, CA, USA): proliferating cell nuclear antigen (PCNA) at 1:100, Tyrosine Hydroxylase (TH) (clone F-11) at 1:100, Glial Fibrillary Acidic Protein (GFAP) (clone 2E1) at 1:100, and Myelin Basic Protein (MBP) (clone F-6) at 1:100. All sections were studied under a brightfield light microscope (Eclipse E600, Nikon, Japan) connected to a digital camera. To quantify the expression intensity of proliferative capacity of the OE, the density of TH-positive neurons, astroglial proliferation, and the expression of MBP, photomicrographs of immunolabelled sections were acquired and imported into ImageJ^®^ Fiji (Schindelin et al., 2012), an open-source image analysis software. For each animal, three representative fields per region (OE, OBs, and hippocampus) were analysed at 20 × magnification. All image quantifications were performed by a single examiner blinded to the experimental groups, to minimize any potential observer-related bias. To minimize regional variability in the OE, especially due to differences in epithelial thickness along the curved surfaces of the turbinates, analysis was restricted to three sections located in the medial wall of the septal OE, ensuring consistency across samples.

In the OBs, quantification was performed in three sections, within the glomerular layer, which was consistently delineated across animals to maintain the consistency of sampling location. In the hippocampus, quantification was similarly standardized by analysing three matched sections per animal, focusing on the regions CA3 and dentate gyrus (DG). Within the DG, particular attention was given to include both the granule cell layer and the subgranular zone. Image processing followed standardized procedure: image channels were split to obtain individual grayscale images per channel, and a constant intensity threshold, established based on control animals, was applied uniformly to all experimental groups. This consistent thresholding ensured comparability of staining intensity measurements across all samples.

### 2.6 Biochemical assays

To determine the oxidative stress parameters the frozen tissues were homogenized with cold buffer (0.32 mM of sucrose, 20 mM of HEPES, 1 mM of MgCl2, and 0.5 mM of phenylmethyl sulfonylfluoride (PMSF), pH 7.4) using a TissueLyser II (30 Hz for 1 min and 30 s, Qiagen, Hilden, Germany), followed by centrifugation at 15000 × *g* at 4°C for 20 min in a refrigerated centrifuge (PrismR, Labnet International, USA). Supernatants were then collected for biochemical analysis. The biochemical assessment was performed at 30°C using a PowerWave XS2 microplate scanning spectrophotometer (Bio-Tek Instruments, USA) (Félix et al., 2018). All samples were analyzed in duplicate and compared against a reagent blank in the appropriate microplate. Values were normalized to the total protein concentration determined by the Bradford assay (Bradford, 1976) at 595 nm, with bovine serum albumin (BSA) as a standard. The total reactive oxygen species (ROS) were quantified using the fluorescent probe DCFH-DA at excitation and emission wavelengths of 485 nm and 530 nm, respectively (Deng et al., 2009). Superoxide dismutase (SOD) activity was determined by the nitroblue tetrazolium (NBT) reduction generated by the xanthine/xanthine oxidase system at 560 nm (Durak et al., 1993). Catalase (CAT) activity was determined by the reduction in absorbance of hydrogen peroxide at 240 nm (Claiborne, 1985). Glutathione peroxidase (GPx) activity was determined by measuring the oxidation of NADPH to NADP + at 340 nm (Massarsky et al., 2017). Glutathione reductase (GR) activity was determined by measuring the NADPH-dependent reduction of oxidized glutathione (glutathione disulfide, GSSG), by monitoring the decrease in absorbance at 340 nm (Massarsky et al., 2017). Glutathione S-transferase (GST) activity was determined by the increase in absorbance due to the conjugation of 1-chloro, 2, 4-dinitrobenzene (CDNB) with reduced glutathione (GSH) at 340 nm (Habig and Jakoby, 1981). Malondialdehyde (MDA) level, an indicator of lipid peroxidation (LPO), was assayed by the thiobarbituric acid (TBA)-based method at 530-nm (MDA-TBA adducts) and 600 nm (non-specific adducts) wavelengths (Wallin et al., 1993). Glutathione levels (GSH and GSSG) were determined by measuring both the reduced (GSH) and the oxidized (GSSG) states using the fluorochrome ortho-phthalaldehyde (OPA) at 320 nm and 420 nm for excitation and emission wavelengths (Gartaganis et al., 2007). The ratio between GSH and GSSG was calculated as the oxidative-stress index (OSI).

### 2.7 Statistical analyses

Statistical analyses were performed using GraphPad Prism version 7.02 for Windows (GraphPad Software, La Jolla, CA, USA). Data normality was assessed using the Shapiro–Wilk test, and homogeneity of variances with Levene’s test. Outliers were identified using the ROUT method (*Q* = 1%) and excluded when appropriate. When assumptions of normality and homoscedasticity were met, group differences were analysed using one-way ANOVA followed by Tukey’s multiple comparisons test, with data expressed as mean ± standard deviation (SD). One-way ANOVA was used to analyse body weight gain, food and water intake, behavioral tests (buried food test and burrowing test), and biochemical and immunohistochemical parameters. Data from the olfactory habituation/cross-habituation test were analysed using repeated measures ANOVA. When normality assumptions were not met, the Kruskal–Wallis test was applied, followed by Dunn’s correction for multiple comparisons. Statistical significance was set at *p* < 0.05.

## 3 Results

### 3.1 Clinical signs and physiological effects following vanadium exposure

Throughout the experimental protocol, no deaths occurred. All animals maintained normal mental status, response to handling, breathing, and hydration within the parameters considered normal for the species. V-exposed animals exhibited sneezing and epistaxis, which began immediately after the first administration and continued until the end of the study. Additionally, no animals showed signs of pain or distress that required sacrifice before the study concluded. Exposure to V significantly reduced body weight gain in the exposed groups (*F*(2,19) = 29.31, *p* = 0.0001) (Table 1). In contrast, brain weights were not significantly affected (*F*(2,19) = 0.01909, *p* = 0.9811). Additionally, recorded food intake showed no significant differences between groups (*p* > 0.05), indicating similar average consumption across all groups. However, water intake exhibited significant differences, with the highest concentration exposure group consuming more than both the lowest exposure group (*p* = 0.0211) and the control group (*p* = 0.0212).

**TABLE 1:** Summary of the effects of intranasal delivery of two V_2_O_5_ concentrations on body weight gain, brain weight, and food and water intake.

Parameters	Control	182 μg	273 μg
Body weight gain (%)	1.682 ± 1.164	−3.300 ± 1.884^a^	−3.203 ± 0.8877^a^
Brain weight (%g)	0.476 ± 0.039	0.480 ± 0.053	0.477 ± 0.042
Food intake (g/animal/day)	20.107	19.758	20.188
Water intake (ml/animal/day)	32.92	33.02	42.571

Body weight gain and brain weight values are expressed as mean ± SD. The values were evaluated for significance using a one-way ANOVA test followed by a Tukey Multiple Comparisons test.

^a^*p* < 0.001 compared to the control.

### 3.2 Olfactory and behavioral deficits following vanadium exposure

**FIGURE 1:**
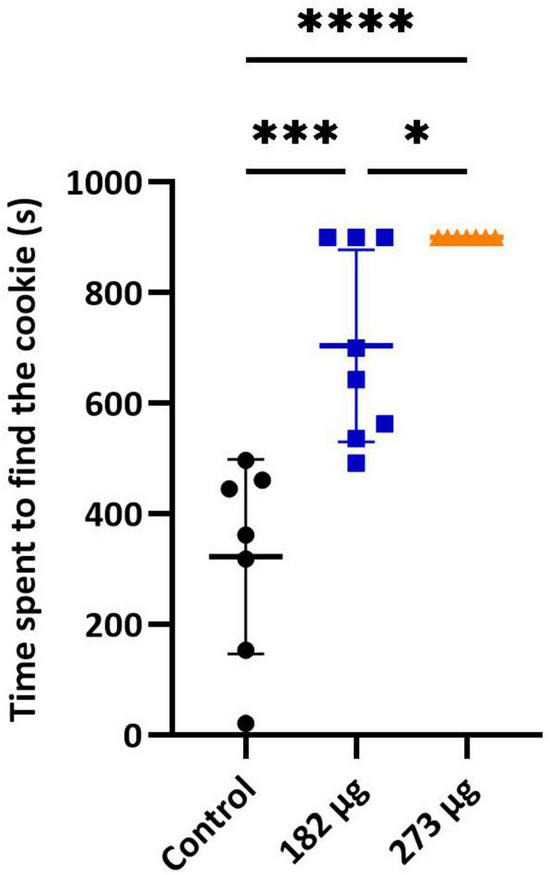
Buried food test. Graph shows the mean ± SD time taken to find the hidden food pellet. Significant differences between groups: *p < 0.05, ***p < 0.001, and ****p < 0.0001.

**FIGURE 2:**
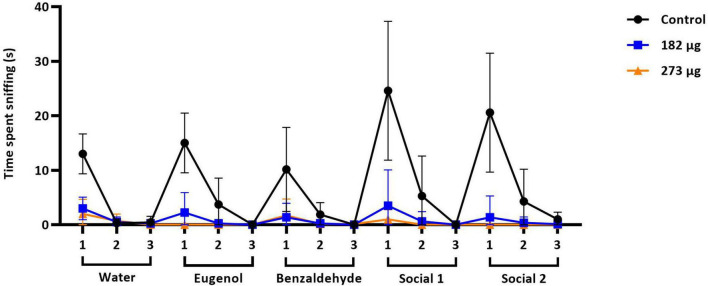
Olfactory habituation/cross-habituation test. The graph shows the mean ± SD olfactory investigation time across three consecutive 2-min trials, each separated by a 1-min inter-trial interval, using water, two non-social, and two social odors.

**FIGURE 3:**
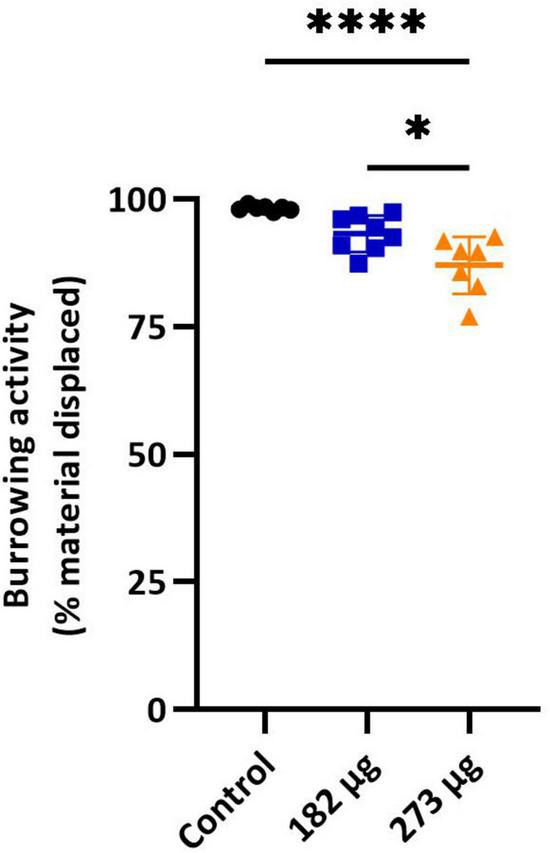
Burrowing test. Graph shows the mean ± SD percentage of cage floor bedding removed from the burrow apparatus by rats in control and treatment groups. Significant differences: *p < 0.05, ****p < 0.0001.

In the buried food test, the intranasal delivery of V had a significant effect on the latency to find the food pellet (*F*(2,19) = 28.92, *p* < 0.0001). *Post hoc* comparisons revealed that the average latency to locate the hidden pellet was significantly longer in the 273 μg (*p* < 0.0001) and 182 μg group (*p* = 0.0002) compared to the control group. Furthermore, a significant difference was found between the two exposed groups (*p* = 0.0430) (Figure 1). Similarly, in the olfactory habituation/cross-habituation test (Figure 2), our findings indicate a significant treatment effect (*F*(2,19) = 49.69, *p* < 0.0001) across all groups regarding the amount of olfactory investigation toward different odors. *Post hoc* analysis revealed that the groups exposed to concentrations of 273 μg and 182 μg spent significantly less time sniffing water and eugenol odors (*p* < 0.001) compared to the control group. Additionally, there were differences in the investigation of social odors. The highest concentration group spent less time sniffing the social 1 (*p* = 0.0062) and social 2 (*p* = 0.0059) odors compared to the control group, while the lowest concentration group also showed reduced sniffing time for social 1 (*p* = 0.0103) and social 2 (*p* = 0.0068) odors compared to the control group. The burrowing test showed that the group exposed to the highest V dose removed significantly less bedding material than the control group (*p* < 0.0001). There was a significant difference between the two exposed groups (*p* < 0.0145), however, the difference between the lowest dose group and the control group was not significant (Figure 3).

### 3.3 Region-specific changes in antioxidant defences without evident oxidative stress following vanadium exposure

**FIGURE 4:**
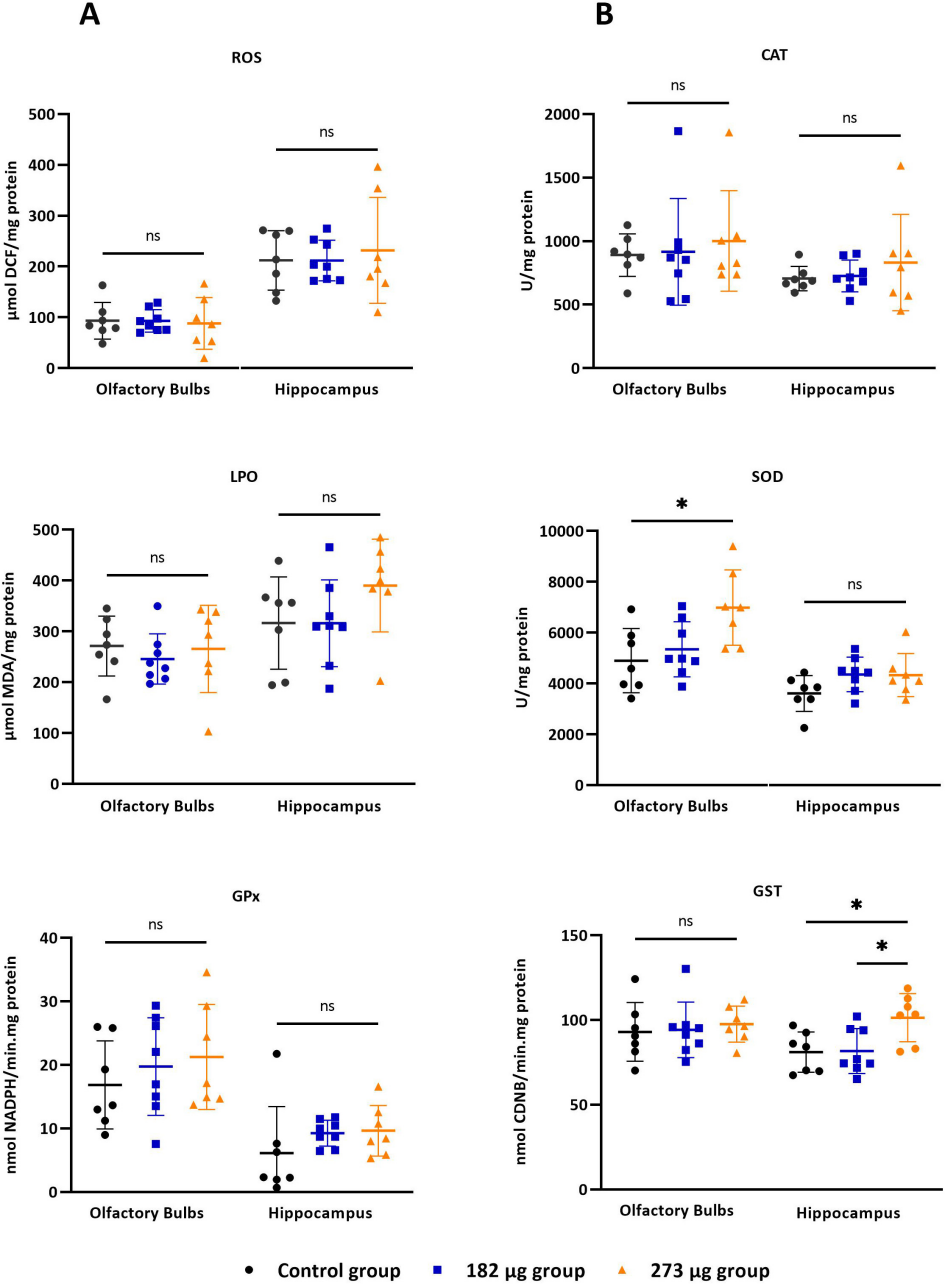
Oxidative stress parameters in the olfactory bulbs and hippocampus following intranasal delivery of 30 μl of vanadium pentoxide solution at concentrations of 273 μg and 182 μg. Parameters assessed: **(A)** Reactive oxygen species (ROS), assessed using the fluorescent probe 2’0.7’.7active oxygen species (ROS), assessed using the fluorescent probe 2’owing intranasal delivery of 30 groups. Significant differences: ther at level 0.05.asal olfact54499/CEECe (GPx) activity. **(B)** Catalase (CAT), superoxide dismutase (SOD), and glutathione S-transferase (GST), with activity determined using 1-chloro-2.4-dinitrobenzene (CDNB) as a substrate. Data are expressed as mean ± standard deviation (SD). Significant differences between groups: *p < 0.05; ns: not significant.

As illustrated in Figure 4 V exposure did not induce significant changes in ROS levels in the OBs (*F*(2,19) = 0.04742, *p* = 0.9538) or hippocampus (*F*(2,19) = 0.1842, *p* = 0.8332) across the exposed groups. In contrast, a significant difference in SOD activity was observed in the OBs (*F*(2,19) = 5.220, *p* = 0.0156), with the highest concentration group showing a significant increase compared to control (*p* = 0.0171). No significant differences were detected in the hippocampus (*F*(2,19) = 2.340, *p* = 0.1234). CAT activity was unaffected in the OBs (*X*^2^(2) = 0.4383, *p* = 0.8184) and hippocampus (*F*(2,19) = 0.5999, *p* = 0.5589) across groups. Similarly, GPx activity showed no significant changes in the OBs (*F*(2,19) = 0.6039, *p* = 0.5568) or hippocampus (*X*^2^(2) = 4.995, *p* = 0.0781). GR activity was unaffected in OBs (*F*(2,19) = 0.2568, *p* = 0.7762), however, in hippocampus there was significant differences between the control and the 182 μg concentration group (*X*^2^(2) = 7.332, *p* = 0.0197). GST activity in the OBs showed no differences (*F*(2,19) = 0.1695, *p* = 0.8453). However, significant differences were observed in the hippocampus (*F*(2,19) = 5.518, *p* = 0.0129) between both exposed groups (*p* = 0.0241) and between the highest concentration group and control group (*p* = 0.0242). GSH (*F*(2,19) = 6.115, *p* = 0.0089) and GSSG (*F*(2,19) = 5.988, *p* = 0.0096) levels in the OBs differed significantly between groups. GSH was significantly increased in the 273 μg group compared to control (*p* = 0.0247) and 182 μg group (*p* = 0.0129), while GSSG was only increased compared to 182 μg group (*p* = 0.0094). However, the associated OSI showed no significant differences (*F*(2,19) = 1.041, *p* = 0.3724).

In the hippocampus, no significant differences were observed in GSH (*F*(2,19) = 2.892, *p* = 0.0801) GSSG (*F*(2,18) = 1.668, *p* = 0.2165), or OSI (*F*(2,17) = 2.853, *p* = 0.0854) among groups. Regarding lipid peroxidation, no significant differences were observed between groups in the OBs (*F*(2,19) = 0.3161, *p* = 0.7327) or in hippocampus (*F*(2,19) = 1.645, *p* = 0.2193).

### 3.4 Histopathological changes in the olfactory system and hippocampus following vanadium exposure

**FIGURE 5:**
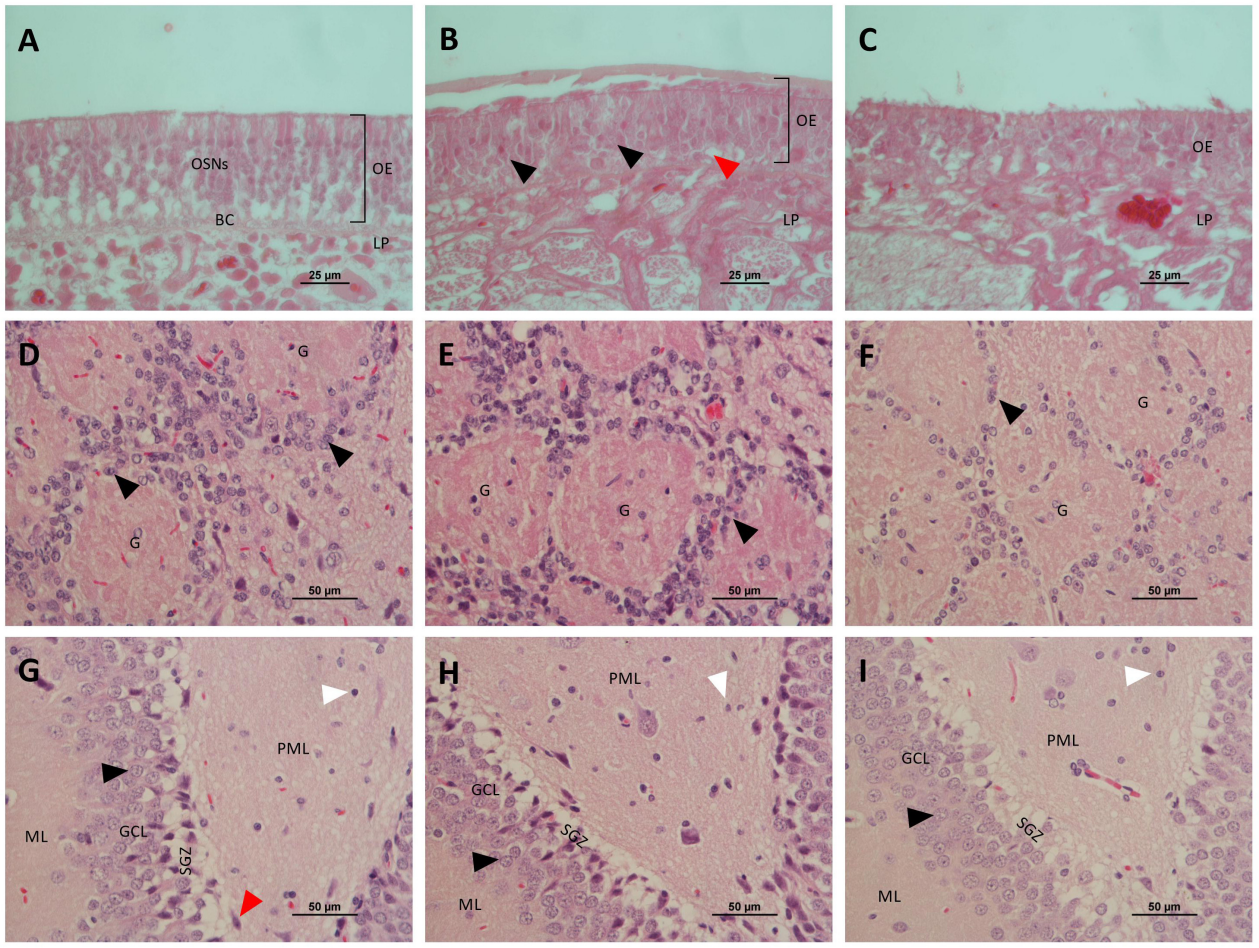
Histological images of the olfactory epithelium, olfactory bulbs, and hippocampus following intranasal exposure to vanadium pentoxide from control, 182 μg, and 273 μg groups (H&E). Olfactory epithelium **(A–C)**: **(A)** Control group: preserved epithelial structure and normal cellular organization of olfactory sensory neurons (OSNs), basal cells (BC), and intact lamina propria (LP); **(B)** 182 μg group: evidence of epithelial disorganization, with vacuolization of sustentacular cells (red arrow) and coagulative necrosis, evident in the epithelium (black arrows) and lamina propria;. **(C)** 273 μg group: loss of normal epithelial architecture and extensive epithelial disruption with pronounced coagulative necrosis. Olfactory bulbs **(D–F)**: **(D)** well-defined glomerular layer, with glomeruli **(G)** appearing as acellular, nearly spherical synaptic structures surrounded by small juxtaglomerular cells (black arrows); **(E)** 182 μg group: glomerular layer showing a reduced density of G, which appear diminished in size when compared to the control; **(F)** 273 μg group: reduced G number and pronounced structural disorganization, associated with decreased juxtaglomerular cells (black arrow) presence. Hippocampus **(G–I)**: **(G)** Control group: normal neuronal structure in the dentate gyrus, showing the molecular layer (ML), the granule cell layer (GCL), composed of densely packed granule cells (black arrow), subgranular zone (SGZ) with immature neurons, and the polymorphic layer (PML) containing pyramidal cells (red arrow) and glial cell nuclei (white arrow); **(H)** 182 μg group: dentate gyrus showing reduced granule cell (black arrow) density in the granule cell layer (GCL) and increased number of glial cells (white arrow) in the polymorphic layer (PML); **(I)** 273 μg group: dentate gyrus showing reduced granule cell (black arrow) density in the granule cell layer (GCL), subgranular zone (SGZ) with sparse immature neurons, and increased number of glial cells (white arrow) in the polymorphic layer (PML).

Histological examination of the nasal cavity revealed that both doses of V exposure caused coagulative necrosis of the respiratory and OE, vacuolization of sustentacular cells, histiocytic infiltration, and a significant decrease in neuronal cell density in the OE, indicating a marked loss of OSNs. In the OBs of the 273 μg and 182 μg vanadium-exposed groups, there was significant evidence (Table 2) of disrupted cellular organization of the olfactory nerve layer, decreased number and size of glomeruli in the glomerular layer, as well as a decreased number of mitral cells, and gliosis compared to the control group. In the hippocampus, both treatment groups exhibited significant differences, with gliosis and neuronal death observed across the different regions (CA1, CA2, CA3, and dentate gyrus) compared to the control group (Figure 5).

**TABLE 2:** Histological changes observed in the different layers of the olfactory system and in the hippocampus after the intranasal delivery of 182 μg, and 273 μg of V_2_O_5_.

Group	Lesion degree	Control	182 μ g	273 μ g	*p-*value
Respiratory epithelium	None	100% (7/7)^a^	0% (0/8)^b^	0% (0/7)^b^	0.0001
	Moderate	0% (0/7)^a^	75% (6/8)^b^	42.9% (3/7)^b^	0.011
	Severe	0% (0/7)	25% (2/8)	57.1% (4/7)	0.065
Olfactory epithelium	None	100% (7/7)^a^	0% (0/8)^b^	0% (0/7)^b^	0.0001
	Moderate	0% (0/7)^a^	75% (6/8)^b^	14.3% (1/7)^a^	0.004
	Severe	.0% (0/7)^a^	25% (2/8)^a^	85.7% (6/7)^b^	0.003
Olfactory bulbs	None	100% (5/5) ^a^	0% (0/7)^b^	0% (0/5)^b^	0.0001
	Moderate	0% (0/5)	57.1% (4/7)	20% (1/5)	0.112
	Severe	0% (0/5)^a^	42.9% (3/7)^a^, ^b^	80% (4/5)^b^	0.034
Hippocampus	None	100% (7/7) ^a^	0% (0/8)^b^	0% (0/7)^b^	0.0001
	Moderate	0% (0/7) ^a^	87.5% (7/8) ^b^	42.9% (3/7) ^a^, ^b^	0.003
	Severe	0% (0/7)	12.5% (1/8)	57.1% (4/7)	0.044

^a,b^Each subscript letter denotes a subset of concentration categories whose proportions do not differ significantly from each other at level 0.05.

#### 3.4.1 Increased proliferative activity in the olfactory epithelium

**FIGURE 6:**
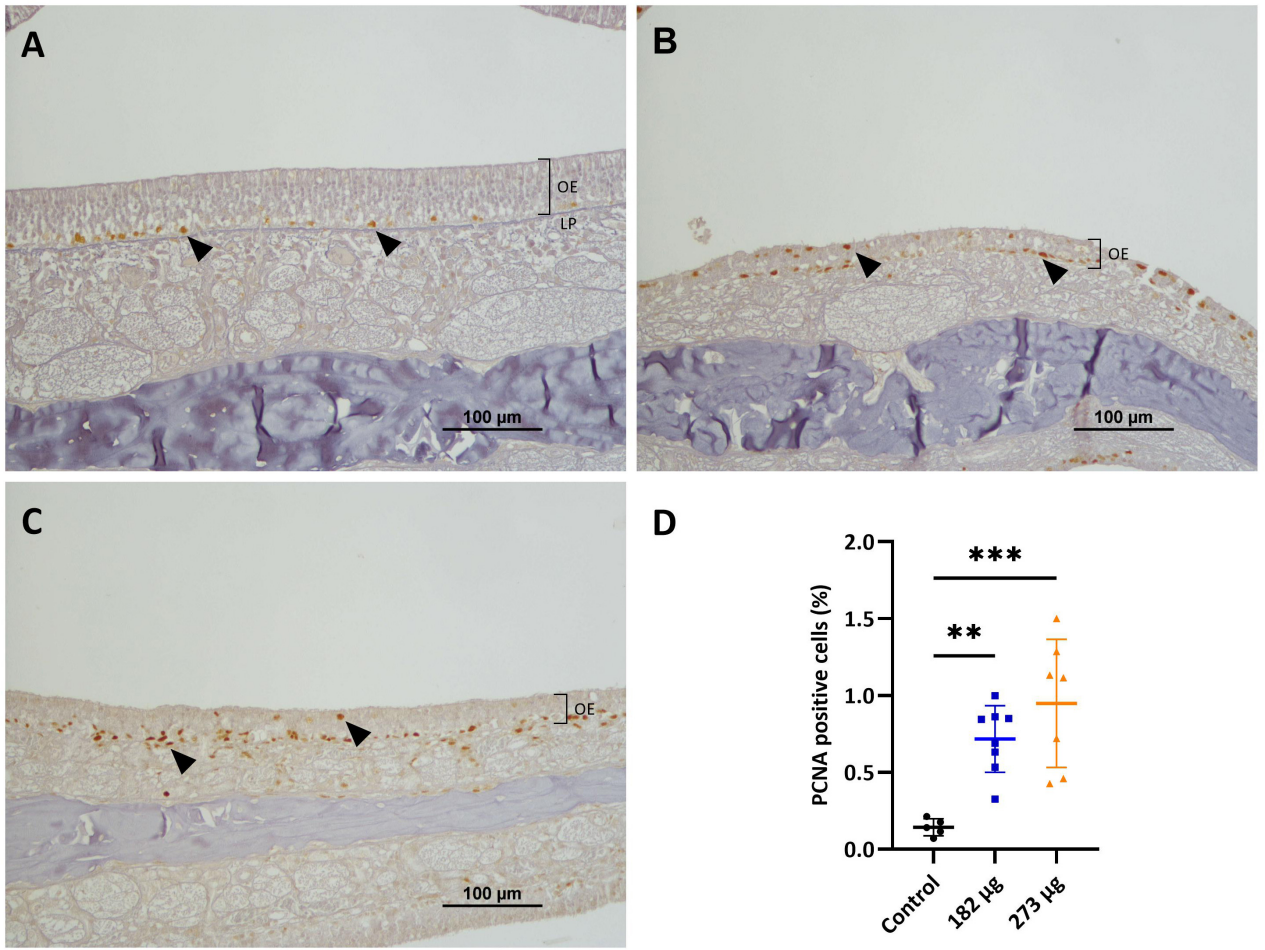
Microscopic images displaying the effects of intranasally administered vanadium pentoxide on cell proliferation in the olfactory epithelium (OE), assessed by proliferating cell nuclear antigen (PCNA) immunohistochemistry. **(A)** Control: limited PCNA immunoreactivity, with positively stained nuclei corresponding to proliferating cells in the basal layer of the OE (black arrows); **(B–C)** 182 μg and 273 μg groups: increased PCNA-positive nuclei (black arrows) throughout the OE, indicating elevated proliferation of basal cells, olfactory sensory neurons (OSNs), and supporting cells, more pronounced at 273 μg. Notice the reduced thickness of the epithelium layer compared to the control group; **(D)** Quantification of PCNA-positive cells in the OE. Data are expressed as mean ± standard deviation (SD). Significant differences between groups: **p < 0.01, ***p < 0.001.

Proliferating cell nuclear antigen quantification revealed significantly increased proliferative activity in the OE of V-exposed groups (*F*(2,17) = 11.94, *p* = 0.0006), with a marked rise in PCNA-positive cells in 273 μg (*p* = 0.0004) and 182 μg (*p* = 0.0069) groups compared to control (Figure 6D). PCNA expression was mainly observed in the nuclei of basal cells, with a smaller number olfactory and supporting cells also showing PCNA positivity (Figures 6A–C).

#### 3.4.2 Decreased tyrosine hydroxylase expression in the olfactory bulb

**FIGURE 7:**
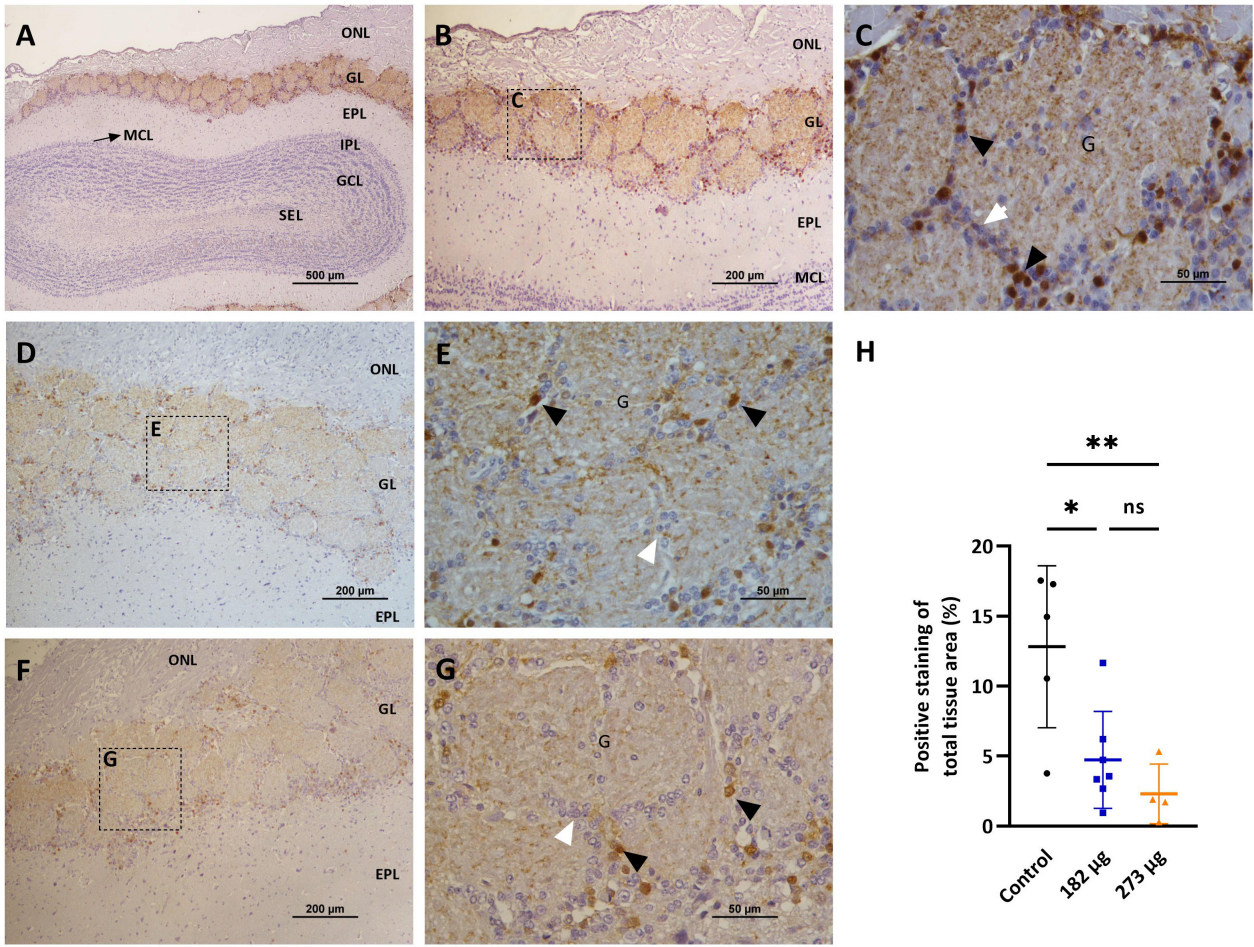
Microscopic images showing the effects of intranasally administered vanadium pentoxide on the olfactory bulbs, assessed by Tyrosine Hydroxylase (TH) immunostaining. **(A–C)** Control group: **(A)** Representative 4 × image of a coronal section of the olfactory bulb, showing TH immunoreactivity and anatomical organization: olfactory nerve layer (ONL), glomerular layer (GL), external plexiform layer (EPL), mitral cell layer (MCL), internal plexiform layer (IPL), granule cell layer (GCL), and subependymal layer (SEL); **(B)** Representative image of the olfactory bulb showing abundant TH-positive dopaminergic neurons in the glomerular layer (GL); **(C)** Enlarged view of the boxed area in panel B, highlighting the glomerular layer (GL), with glomeruli **(G)**, surrounded by juxtaglomerular cells (white arrows), along with TH-positive dopaminergic neurons (black arrows); **(D–G)** Representative images of the olfactory bulb from vanadium pentoxide-exposed groups: **(D–E)** 182 μg and **(F–G)** 273 μg. **(D–F)** Low-magnification images of the olfactory bulb showing the olfactory nerve layer (ONL), glomerular layer (GL), and external plexiform layer (EPL). Boxed regions indicate areas selected for higher magnification in panels E and G; **(E,G)** Enlarged views of the boxed regions show reduced density and intensity of TH-positive dopaminergic neurons (black arrows) and surrounding juxtaglomerular cells (white arrows) within the glomeruli **(G)**. The reduction appears more pronounced in the 273 μg group; **(H)** Quantification of TH expression in the olfactory bulb. Data are expressed as mean ± standard deviation (SD). Significant differences between groups: *p < 0.05, **p < 0.01; ns, not significant.

**FIGURE 8:**
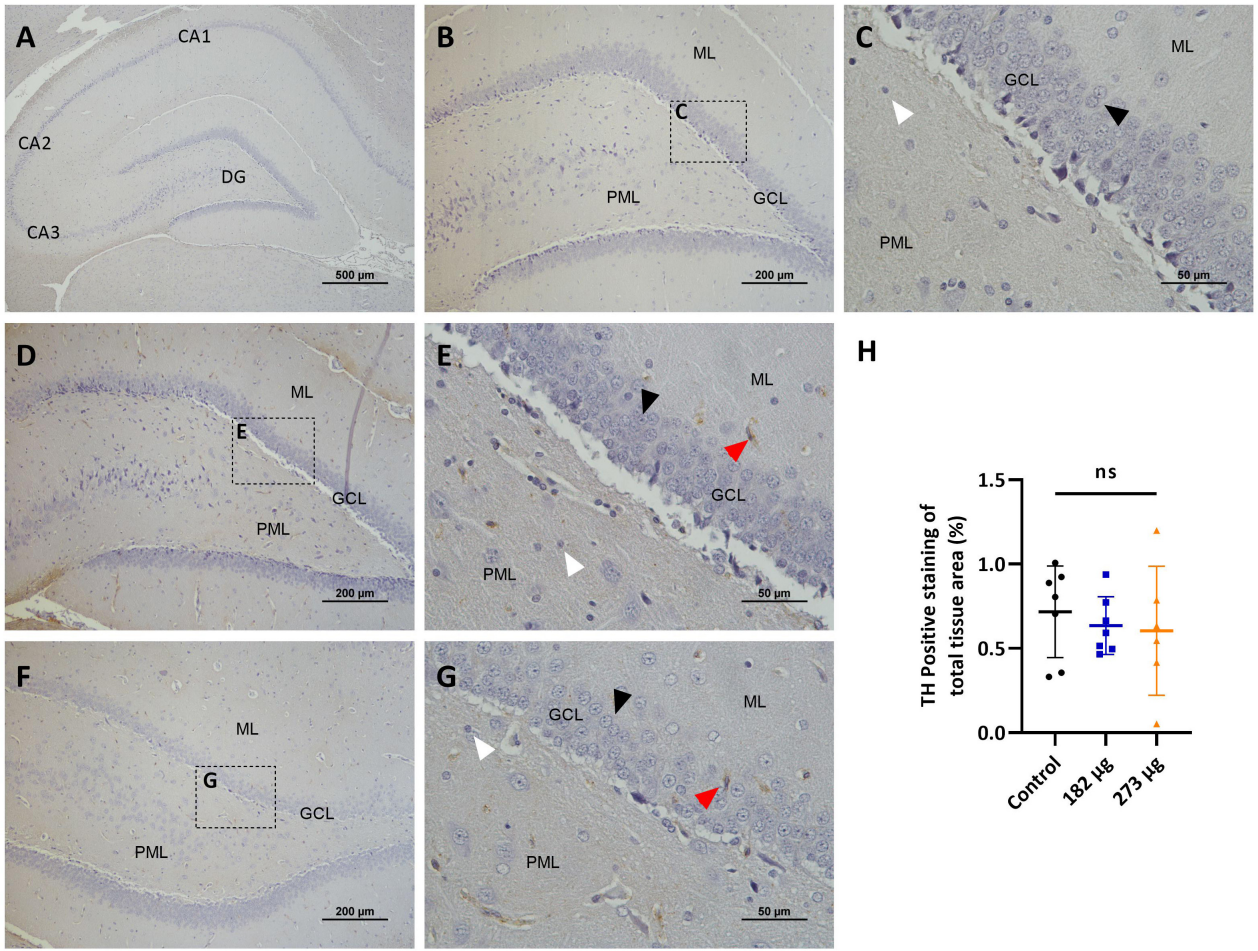
Microscopic images depicting the effects of intranasally administered vanadium pentoxide in the hippocampus, assessed by Tyrosine Hydroxylase (TH) immunostaining. **(A–C)** Control group: **(A)** Low-magnification (4 ×) section of the hippocampus showing its anatomical organization, including the C-shaped structure of the *Cornu Ammonis* regions (CA1, CA2, CA3) and the dentate gyrus (DG); **(B)** Higher-magnification image of the dentate gyrus showing minimal or absent TH immunoreactivity across distinct layers: molecular layer (ML), granule cell layer (GCL) and polymorphic layer (PML); **(C)** Enlarged view of the boxed region in panel B, highlighting the three distinct layers of the dentate gyrus: molecular layer (ML), granule cell layer (GCL) composed of densely packed granule cells (black arrow) and polymorphic layer (PML), with some glial cells (white arrow); **(D–G)** Representative images of the dentate gyrus from vanadium pentoxide-exposed groups: **(D–E)** 182 μg and **(F–G)** 273 μg; **(D,F)** Low-magnification images showing the anatomical organization of the dentate gyrus, including the molecular layer (ML), granule cell layer (GCL), and polymorphic layer (PML); **(E,G)** Enlarged views of the boxed areas in panels **(D,F)**, respectively. As in the control group, TH immunoreactivity remains minimal or absent. Occasional TH-positive neurons (red arrows) can be seen, but are rare and scattered. Granule cells (black arrows) and glial cells (white arrows) are also identified; **(H)** Quantification of TH expression in the hippocampus. Data are presented as mean ± standard deviation (SD). Differences between groups, ns, not significant.

In the OBs, the control group shows intense TH immunostaining, clearly visible in the neurons of the glomerular layer, in a perinuclear position, and also in some cellular processes (dendrites and axons) of the external plexiform layer (EPL) (Figures 7A–C). The V treated groups exhibit a significant decrease in TH expression in the intensity and extent (*F*(2, 13) = 8.626, *p* = 0.0041) more evident in highest concentration group (*p* = 0.0057), and in the lowest (*p* = 0.0132), when compared to the control group (Figures 7D–H). In the hippocampus, no significant immunostaining of neuronal cells was observed, with few positive neurons visible in the control group and in the vanadium treated groups (Figures 8A–H).

#### 3.4.3 Astrocyte migration and proliferation in the olfactory bulb and hippocampus

**FIGURE 9:**
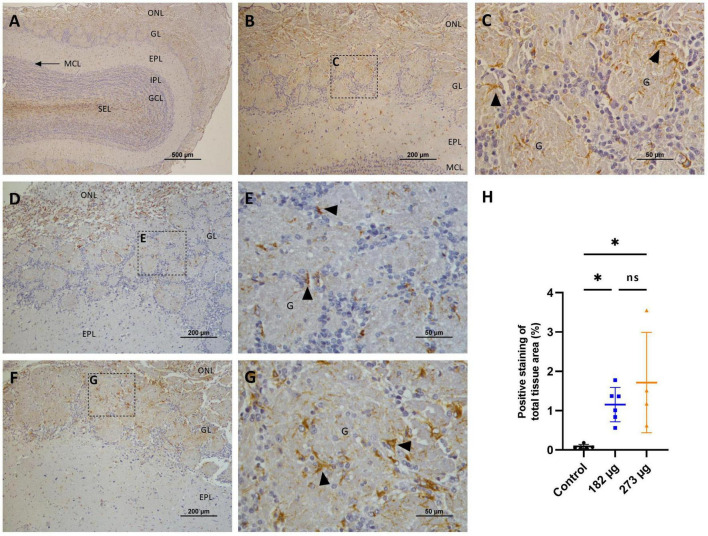
Microscopic images illustrating the effects of intranasally administered vanadium pentoxide in the olfactory bulbs, assessed by Glial Fibrillary Acidic Protein (GFAP) immunostaining. **(A–C)** Control group: **(A)** Low-magnification (4 ×) coronal section of the olfactory bulb showing GFAP immunoreactivity and anatomical organization: olfactory nerve layer (ONL), glomerular layer (GL), external plexiform layer (EPL), mitral cell layer (MCL), internal plexiform layer (IPL), granule cell layer (GCL), and subependymal layer (SEL); **(B)** Representative image of OB highlighting GFAP-positive astrocytes in the glomerular layer (GL) and external plexiform layer (EPL); **(C)** Enlarged view of the boxed region in panel B, showing GFAP-positive astrocytes (arrows) with characteristic star-shaped morphology, distributed within and around the glomeruli **(G)** in the glomerular layer (GL); **(D–G)** Vanadium pentoxide exposed groups: **(D–E)** 182 μg and **(F–G)** 273 μg: **(D,F)** Low-magnification images of the olfactory bulb showing the olfactory nerve layer (ONL), glomerular layer (GL), and external plexiform layer (EPL). Boxed regions indicate areas selected for higher magnification in panels E and G; **(E,G)** Higher-magnification images of the boxed regions in panels D and F, respectively, highlighting a marked accumulation of GFAP-positive astrocytes (arrows) within and surrounding the glomeruli **(G)**; **(H)** Quantification of GFAP expression in the olfactory bulb. Data are expressed as mean ± standard deviation (SD). Significant differences between groups *p < 0.05; ns, not significant.

**FIGURE 10:**
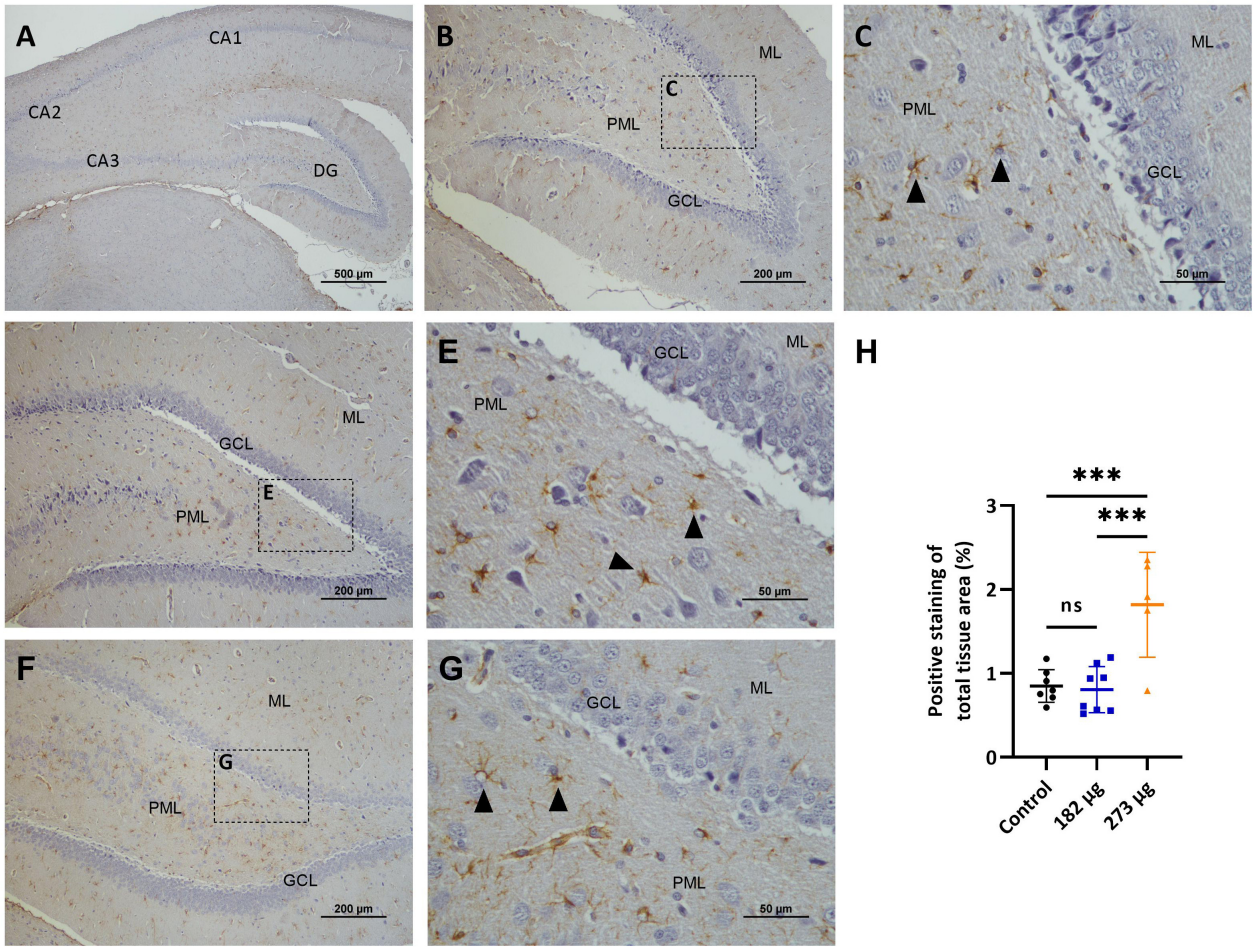
Microscopic images displaying the effects of intranasally administered vanadium pentoxide in the hippocampus, assessed by Glial Fibrillary Acidic Protein (GFAP) immunostaining. **(A–C)** Control group: **(A)** Low-magnification (4 ×) section of the hippocampus showing the anatomical organization, including the characteristic C-shaped structure of the *Cornu Ammonis* regions (CA1, CA2, CA3) and the dentate gyrus (DG); **(B)** Representative higher-magnification image of the dentate gyrus (DG), showing GFAP-positive astrocytes distributed throughout the molecular layer (ML), granule cell layer (GCL), and polymorphic layer (PML); **(C)** Enlarged view of the boxed region in panel B, highlighting the three distinct layers of the dentate gyrus: molecular layer (ML), granule cell layer (GCL) and polymorphic layer (PML), with GFAP-positive astrocytes (arrows); **(D–G)** Vanadium pentoxide exposed groups: **(D–E)** 182 μg and **(F–G)** 273 μg. **(D–F)** Low-magnification images of the hippocampus, showing the anatomical organization of the dentate gyrus, including the molecular layer (ML), granule cell layer (GCL), and the molecular layer (ML). Boxed areas indicate regions selected for higher-magnification. **(E,G)** Higher-magnification views of the boxed areas in panels **(D,F)**, respectively. GFAP-positive astrocytes (arrows) are observed in the molecular layer (ML) and the polymorfic layer (PML) of the dentate gyrus; **(H)** Quantification of GFAP expression in the hippocampus. Data are expressed as mean ± standard deviation (SD). Significant differences between groups ***p < 0.001; ns, not significant.

The GFAP immunostaining revealed a significant increase in astroglial proliferation in the glomerular layer in the OBs of both V exposed groups (H (2, 17) = 8.187 *p* = 0.0114) compared with control group (Figures 9A–H). In the hippocampus, GFAP expression was significantly increased only in the 273 μg concentration group (*F*(2, 17) = 13.57, *p* = 0.0003) compared to the 182 μg concentration group and the control group, in the CA1, CA2, CA3 and dentate gyrus regions. No significant differences were observed between the 182 μg concentration group and the control group (Figures 10A–H).

#### 3.4.4 Preservation of myelin integrity in the olfactory bulb and hippocampus

**FIGURE 11:**
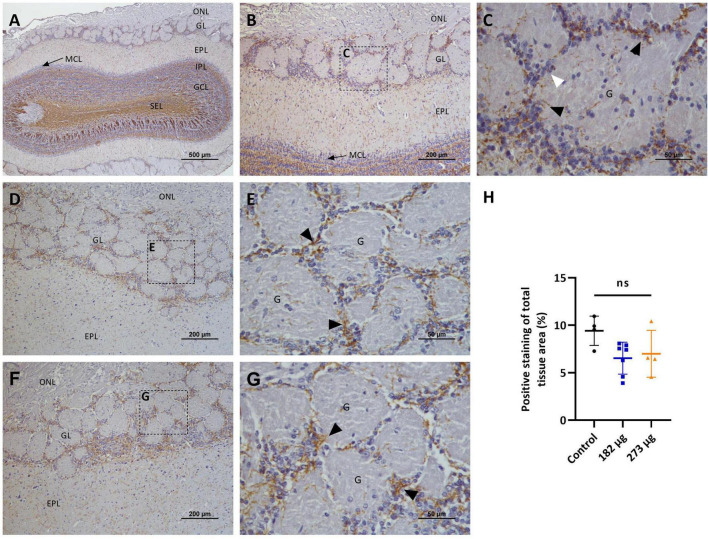
Microscopic images showing the effects of intranasally administered vanadium pentoxide in the olfactory bulbs (OB), assessed by myelin basic protein (MBP) immunostaining. **(A–C)** Control group: **(A)** Low-magnification (4 ×) coronal section of the olfactory bulb showing the distribution of MBP-immunoreactive myelin fibers across different olfactory bulb layers: olfactory nerve layer (ONL), glomerular layer (GL), external plexiform layer (EPL), mitral cell layer (MCL), internal plexiform layer (IPL), granule cell layer (GCL), and subependymal layer (SEL); **(B)** Higher-magnification image of the OB highlighting the MBP- immunoreactive fibers in olfactory nerve layer (ONL), glomerular layer (GL), external plexiform layer (EPL) and mitral cell layer (MCL); **(C)** Enlarged view of the boxed region in panel B, showing compact and well-organized MBP-immunoreactive fibers (black arrows) surrounding the glomeruli **(G)** and closely associated with juxtaglomerular cells (white arrow) in the glomerular layer (GL); **(D–G)** Vanadium pentoxide-exposed groups: **(D–E)** 182 μg and **(F–G)** 273 μg. **(D,F)** Low-magnification images of the OB showing the olfactory nerve layer (ONL), glomerular layer (GL), and external plexiform layer (EPL); Boxed areas indicate regions selected for higher magnification. **(E,G)** Higher-magnification views of the boxed areas in panels **(D,F)**, respectively, showing MBP-immunoreactive fibers (black arrows) with a similar distribution and morphology to those observed in the control group; **(H)** Quantification of MBP expression in the olfactory bulb. Data are expressed as mean ± standard deviation (SD). Differences between groups, ns, not significant.

**FIGURE 12:**
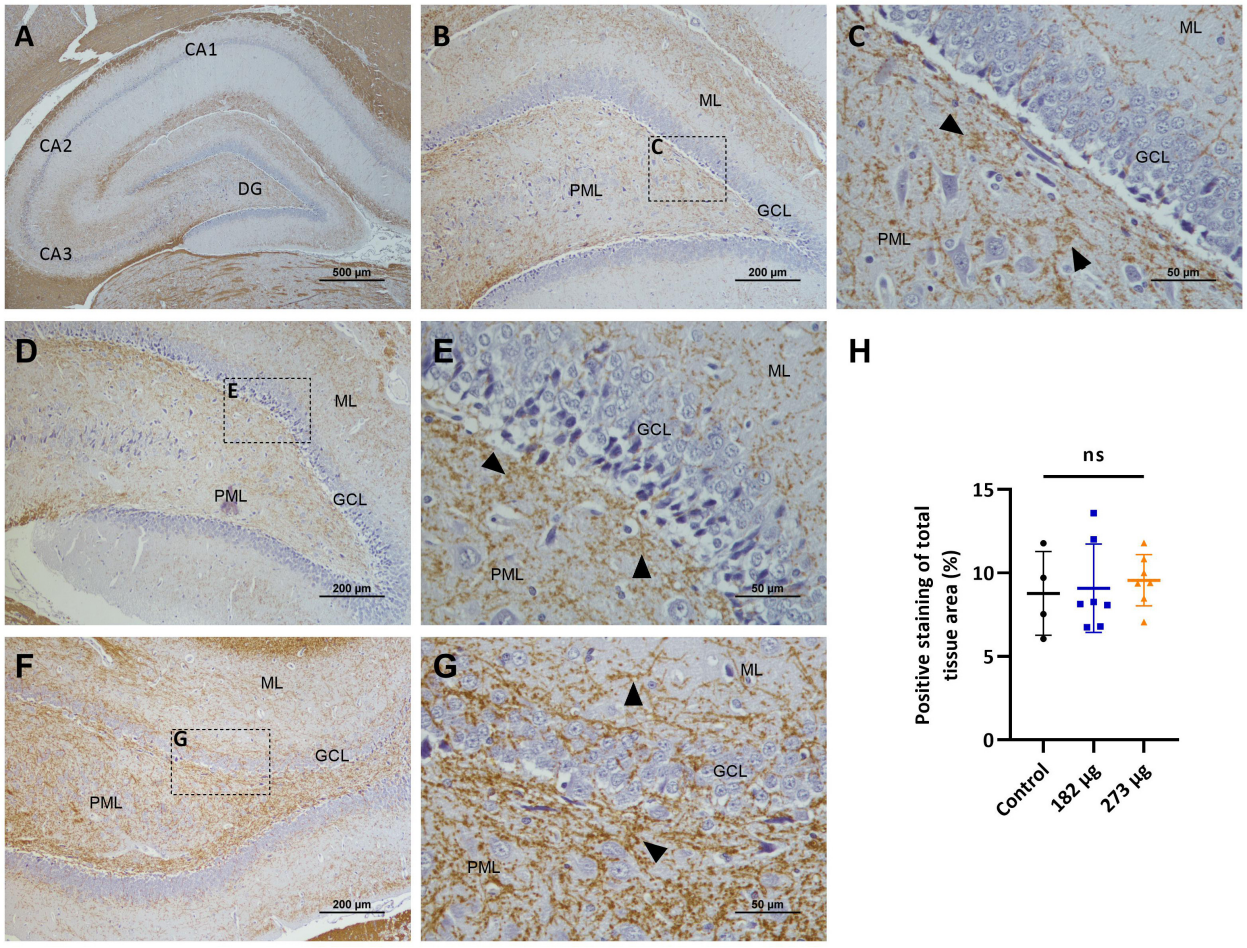
Microscopic images displaying the effects of intranasally administered vanadium pentoxide in the hippocampus, assessed by myelin basic protein (MBP) immunostaining. **(A–C)** Control group: **(A)** Low-magnification (4 ×) section of the hippocampus showing the distribution of MBP-immunoreactive fibers across different anatomical regions CA1, CA2, CA3 and the dentate gyrus (DG); **(B)** Higher-magnification image of the dentate gyrus region showing MBP- immunoreactive fibers distributed along the molecular layer (ML), granule cell layer (GCL), and polymorphic layer (PML); **(C)** Enlarged view of the boxed area in panel B, highlighting compact and well-organized MBP-immunoreactive fibers (arrows) predominantly observed in the molecular layer (ML) and polymorphic layer (PML); **(D–G)** Vanadium pentoxide-exposed groups: **(D,E)** 182 μg group; **(F–G)** 273 μg group. **(D,F)** Low-magnification images showing the general distribution of MBP-immunoreactive fibers in the dentate gyrus. Boxed areas indicate regions selected for higher magnification. **(E,G)** Higher-magnification views of the boxed regions in panels **(D,F)**, respectively, showing MBP-immunoreactive fibers (arrows) with a distribution similar to the control. In animals exposed to 273 μg, subtle alterations are observed, including areas with compact or slightly disorganized fibers (arrows) in the molecular layer (ML) and polymorphic layer (PML); **(H)** Quantification of MBP expression in the hippocampus. Data are presented as mean ± standard deviation (SD). Differences between groups, ns: not significant.

The immune-histochemical analysis using anti-MBP antibody in the OBs revealed abundant expression of this protein in the cellular processes of glomerular neurons, in the axons present in the external plexiform layer, and also in the granular layer (Figures 11A–H). In the hippocampus, expression is similarly abundant in the CA1, CA2, CA3 regions and in dentate gyrus (Figures 12A–H). In terms of extent and intensity, no significant differences in MBP expression were observed in the OB (*F*(2, 12) = 3.150, *p* = 0.0795) (Figure 11H) or hippocampus (*F*(2, 15) = 0.1748, *p* = 0.8413) (Figure 12H) of any groups exposed to V_2_O_5_. However, some alterations in the expression were noted in animals exposed to the highest concentration, particularly in the glomerular layer of the OB. In this region, microscopic examination revealed focal disruptions of the myelin sheath architecture, characterized by segmental thinning, discontinuities, and areas of decreased compaction, suggestive of demyelination or incomplete myelination (Figures 11F, G).

## 4 Discussion

In this study, we evaluated, for the first time to our knowledge, the effects of NTB delivery of two distinct doses of V_2_O_5_ on the olfactory system in a rat model. Unlike previous research, which primarily focused on V’s toxicity in specific brain areas or its systemic effects through different exposure routes, our study uniquely explores its impact on the NTB pathway following intranasal delivery. Using a novel approach, we inserted a cannula through the nasal cavity to directly target the olfactory region and, consequently, the OB. By analysing the toxic responses in the RE, OE, OB, and hippocampus, our research aims not only to provide new insights into V’s direct effects but also to characterize NTB delivery in an animal model. Additionally, it seeks to fill an important gap in understanding how intranasal delivery of different substances or similar approaches can influence the NTB pathway, an essential route due to its direct connection to the CNS. Therefore, we consider a detailed characterization of this animal model crucial for validating future results in studies on olfactory dysfunction and for providing information that may enhance the translation of findings to the target species. The V doses used in this study were chosen to reflect environmentally and occupationally relevant exposures, ensuring the induction of detectable neurotoxic effects. The dose of 182 μg V_2_O_5_ was selected based on the work of Ngwa et al. (2014), who reported that this amount falls within a range consistent with estimated human V accumulation through inhalation. According to their review, individuals in rural environments may retain approximately 100 μg of V, while those in urban or industrial areas may accumulate between 142 and 570 μg, with values exceeding 5700 μg in high-pollution or occupational contexts. The dose of 273 μg V_2_O_5_, also within the estimated urban-industrial exposure range, was defined based on preliminary trials conducted prior to the experimental protocol. These trials aimed to identify a higher concentration capable of producing histological and behavioral alterations in the olfactory and hippocampal regions without compromising animal welfare. Together, these two doses allowed us to assess a spectrum of neurotoxic effects, from early-stage impairments to more severe damage, thus increasing the translational value of our model.

From an animal welfare perspective, intranasal exposure to V_2_O_5_ at the tested concentrations did not lead to major clinical signs of distress. Although a significant reduction in body weight gain was observed in the exposed groups, food intake remained stable across all groups, suggesting that the weight changes were not related to reduced appetite. Water intake was significantly increased in the higher dose group, possibly reflecting a physiological response to the exposure. Additionally, aside from occasional sneezing and mild epistaxis, no other alterations were recorded in the welfare score sheet parameters. Supporting these findings, García et al. (2004) 2005, who administered vanadium intraperitoneally, did not observe signs of vanadium toxicity, with treated rats only showing piloerection. In contrast, Sun et al. (2017) observed redness in the mouth and nose, drowsiness, and decreased activity in rats exposed to higher doses of vanadium via the oral route. Similarly, Rajendran et al. (2016), reported increased severity of toxicity signs, including hypoactivity and mortality, in a whole-body inhalation study as vanadium concentration increased. These findings suggest that the effects of vanadium on animal welfare may vary depending on factors such as the oxidation state, solubility, bioavailability, dose, and route of administration of the vanadium compounds.

The body weight gain in the V-treated rats was significantly reduced due to exposure, consistent with the weight loss observed in some studies (Sasi et al., 1994; Haider et al., 1998; Sanchez et al., 1998; Cuesta et al., 2011). However, other studies have reported no significant changes in body weight following V exposure (García et al., 2004, 2005; Montiel-Flores et al., 2021). Regarding brain weight, no differences were observed between the groups. These findings suggest that the effects of V exposure may vary depending on the route of administration, experimental conditions, and dosages used. Although reduced food intake is a common effect of V exposure (Wang et al., 2001), no decrease in food ingestion was observed in the V-treated rats in our study. Water intake in our study was higher in the rats exposed to the highest concentration of V. However, some studies have reported no significant differences in water consumption between exposed and control groups, and at least one study has observed a decrease in water intake in vanadium-exposed rats compared to controls (Espinosa-Zurutuza et al., 2018).

### 4.1 Changes in the nasal epithelium

It is well-established that certain metals can damage the nasal epithelium (Sunderman, 2001). In the present study, we observed coagulative necrosis of the respiratory and olfactory epithelial layers, loss of OSNs, vacuolization of sustentacular cells, and histiocytic infiltration in both V-exposed groups, with these alterations being more pronounced in the group exposed to the higher concentration. To our knowledge, nasal pathology resulting from V exposure has not been previously characterized in rodents following intranasal instillation, making this one of the novel contributions of our work. While prior studies have described V-induced neurotoxicity in the OB and associated olfactory deficits (Ngwa et al., 2014; Colín-Barenque et al., 2015, 2018), detailed histopathological analysis of the OE was lacking. Our data provide the first *in vivo* evidence of dose-dependent damage to the OE, supported by increased PCNA expression. PCNA, a nuclear protein predominantly expressed during the S phase of the cell cycle, is a well-established marker of cell proliferation. The upregulation of PCNA positive cells, particularly in basal layers, suggests a regenerative response in the OE following V-induced injury. This proliferative activity likely reflects an attempt to restore epithelial integrity, consistent with the OE’s known capacity for regeneration after toxic insult (Yu and Wu, 2017). However, the presence of OE thinning and OSN loss, despite this response, indicates an active but insufficient repair mechanism proportional to the severity of damage. These findings expand current knowledge by demonstrating that V toxicity affects not only central, but also peripheral components of the olfactory system In addition, human studies on individuals with occupational exposure to V report irritation of the upper respiratory tract, along with histopathological changes such as sub epithelial zones of round cell infiltration in the lamina propria and hyperemia (Kiviluoto et al., 1979). Similarly, several inhalation studies with other toxic metals, including manganese (Dorman et al., 2004; Foster et al., 2018), zinc sulfate (Burd, 1993), and zinc oxide (Gao et al., 2024), have reported comparable pathological changes in the nasal epithelium. Importantly, while our findings clearly demonstrate peripheral damage, V’s known ability to cross the BBB following systemic exposure suggests that central structures, including the OBs, may also be affected. This dual potential complicates the interpretation of the observed olfactory impairments, which may arise from both disrupted odor detection at the periphery and central neurotoxic effects. Such a combined mechanism is consistent with other toxicological and viral models (Ye et al., 2021), where olfactory dysfunction reflects combined peripheral and central damage. Although we did not assess V accumulation in the brain, our results underscore the need for future studies to determine the relative contribution of each site. Altogether, our findings document previously undescribed V-induced nasal pathology and highlight the need to investigate the full extent of the olfactory pathway’s vulnerability.

### 4.2 Alterations in the olfactory bulbs

V exposure has been linked to necrotic neuronal death in the OB, as demonstrated in mice exposed to repeated inhalation of V_2_O_5_ (Colín-Barenque et al., 2015, 2018; Gilbert et al., 2023). In our study, nasal instillation of V similarly resulted in structural changes in the OBs, including a reduction in the size and number of glomeruli, necrosis, gliosis, and a marked decrease in mitral cell populations. TH immune-histochemical analysis revealed a significant loss of dopaminergic neurons in the glomerular layer of the OB in both V-exposed groups, highlighting a potential link between V exposure and olfactory deficits. Supporting these findings, histological analyses in other studies have demonstrated a significant loss of TH-positive dopaminergic neurons in the glomerular and granular layers of the OB, with dopaminergic neurons in the glomerular layer being particularly affected by intranasal V exposure (Ngwa et al., 2014, 2024). This loss of TH-positive neurons has also been observed in the substantia nigra, suggesting that V toxicity induces catecholaminergic alterations. These effects may result from the inhibition of key phosphoryl transfer reactions critical for regulating neuronal function, with observed disruptions potentially contributing to neurodegeneration and behavioral impairments (Avila-Costa et al., 2004).

In addition to the loss of dopaminergic neurons, we observed a significant accumulation of astrocytes in the glomerular layer of the OB in both exposed groups, as evidenced by GFAP immunostaining. Astrogliosis following V exposure has been previously reported in various CNS regions (García et al., 2005; Azeez et al., 2016; Igado et al., 2020). However, similar astrocytic activation in the OB has only been documented by Ngwa et al. (2014) 2024. The observed astrogliosis suggests a neuro-inflammatory process, since microglia activation and astrocytic proliferation are characteristic in CNS lesions (Azeez et al., 2016). Microglia, the brain’s resident immune cells, are activated early in demyelination, a process in which astrocytes not only contribute to microglia recruitment but may also inhibit remyelination (Azeez et al., 2016). Consistent with these, our MBP immune-histochemical analysis revealed disrupted and reduced myelin sheaths, particularly within the external plexiform layer of OBs exposed to the higher concentration, potentially secondary to toxicant-induced oligodendrocyte dysfunction or neuroinflammatory processes (Todorich et al., 2011). Specific data on V’s effects on OB myelin are limited, however, several studies have reported that V disrupts myelin formation in different brain regions of rats and mice exposed during early development (Soazo and Garcia, 2007; Mustapha et al., 2014). Research has further shown that V exposure can initiate peroxidative reactions across brain regions, reducing fatty acids, altering lipid profiles resulting in protein loss (Sasi et al., 1994). Similarly, García et al. (2004) reported that exposure to sodium metavanadate decreased MBP immunostaining in affected brain regions. Given MBP’s critical role in forming multi-layered myelin, this reduction suggests that V disrupts essential MBP-lipid interactions, hindering myelin formation and maintenance (García et al., 2004). Although qualitative histological observations indicated potential alterations in myelin integrity, quantitative analysis of MBP immunolabelling did not reach statistical significance. This discrepancy may reflect some limitations of immunohistochemistry in detecting subtle changes in protein expression, particularly when compared to more sensitive techniques such as Western blotting. Future studies incorporating complementary approaches like Western blotting could provide more definitive quantitative insight into MBP levels.

Oxidative stress induced by V exposure is characterized by the generation of ROS and LPO, which contribute to cellular and neuronal damage. While most research has focused on V’s effects in other brain regions, its impact on the OBs remains unexplored. To bridge this gap, our study assessed oxidative stress markers, including ROS and LPO, as well as the activity of key antioxidant enzymes such as SOD, GPX, GST, and CAT. Our results showed that after 4 weeks of exposure to 273 μg and 182 μg of V_2_O_5_, there were no significant differences in ROS and LPO levels between the exposed groups and the control. However, a slight increase in these markers was observed in the V-exposed groups, being more pronounced at the higher concentration. The slight elevation in ROS and MDA levels may be attributed to the upregulated activity of antioxidant enzymes, particularly SOD. This enzyme is crucial in neutralizing superoxide radicals (O_2_-) produced during V metabolism, converting them into hydrogen peroxide (H_2_O_2_), which CAT and GPx further process to limit ROS accumulation. In this study, SOD activity increased in both V-exposed groups, with a significant rise in the higher concentration group, suggesting that this response may have helped to mitigate ROS accumulation. These findings differ from previous studies where V exposure led to significant increases in ROS and MDA levels in the OBs of mice (Colín-Barenque et al., 2018) and rats (Igado et al., 2012), as well as in other brain regions (García et al., 2004; Igado et al., 2012; Folarin et al., 2017; Adebiyi et al., 2019), alongside a decline in brain SOD activity (Folarin et al., 2017; Adebiyi et al., 2019). Regarding CAT, GST, and GPx activities, no significant differences were observed between the exposed groups and the control group. This suggests that, despite exposure, these enzymes maintained stable activity levels. GPx, one of the most abundant antioxidants in the brain, plays a vital role in preventing the formation of peroxyl and hydroxyl radicals. Its activity indicates an effective enzymatic response that prevents H_2_O_2_ accumulation maintaining oxidative balance. Compared with other organs, the brain has lower levels of CAT, so its role in neutralizing H_2_O_2_ is often complemented by GPx. In V-exposed models, unchanged CAT activity could suggest that the oxidative stress remained within manageable limits, potentially due to compensatory actions by other antioxidant pathways or enzymes (Cuesta et al., 2011). Our results contrast with those reported in previous research that has shown that V inhalation induced significantly increased GPx activity in the OBs, associated with neuronal death, reduced dendritic spine density, and olfactory impairment, likely due to oxidative stress (Colín-Barenque et al., 2015). On the other hand, other studies have reported significant decreases in these enzyme activities following exposure in different brain regions (Igado et al., 2012; Folarin et al., 2017, 2018; Adekeye et al., 2020; Adekeye et al., 2022). GST supports the detoxification of secondary ROS generated when primary ROS interact with cellular components (Folarin et al., 2018; Hubatsch et al., 1998). Unlike studies reporting GST inhibition under V exposure due to GSH depletion or oxidative damage (Folarin et al., 2018), our results showed no significant changes in GST activity in the OBs. The concurrent increase in both GSH and GSSG observed in the OBs at the highest dose suggests sufficient substrate availability and preserved enzymatic function under stress conditions. However, despite these changes, the OSI remained unchanged. This apparent discrepancy may reflect V-specific redox dynamics. As described by He et al. (2022), vanadium undergoes intracellular redox cycling, where V^5 +^ is reduced to V^4 +^, leading to direct oxidation of GSH, stimulation of GSH synthesis, and increased export of glutathione species, without necessarily triggering a classical ROS response. Consequently, the elevated glutathione levels might represent a compensatory adaptation that maintains redox homeostasis. The unchanged OSI, calculated as the ratio of GSSG to GSH, likely results from a proportional increase in both reduced and oxidized forms, suggesting that although glutathione turnover was enhanced, the redox buffering capacity remained effective. This pattern aligns with interpretations that mild or early oxidative challenges can activate protective antioxidant responses without leading to measurable ROS or lipid peroxidation.

### 4.3 Changes in hippocampus

In the hippocampus, both treatment groups exhibited histological alterations, consistent with the observations by Avila-Costa et al. (2006) and Gilbert et al. (2023). Their study showed that V inhalation in mice caused a loss of dendritic spines, necrotic-like cell death, and significant alterations in the CA1 region of the hippocampus. These findings underline the neurotoxic effects of V on hippocampal structures, highlighting its potential to disrupt neuronal networks critical to memory and cognitive functions. The absence of significant TH immunostaining in the hippocampus observed in our study, in control and V-treated groups, suggests a limited presence or involvement of dopaminergic neurons in this region. While the lack of TH immunostaining might seem to exclude a dopaminergic contribution to V-induced hippocampal dysfunction, it remains possible that V exposure indirectly affects dopaminergic signaling in target regions connected to the hippocampus, such as the ventral tegmental area or the prefrontal cortex (Tsetsenis et al., 2021). Future investigations utilizing markers for other neurotransmitter systems or functional assessments of hippocampal circuits could further elucidate these interactions. The significant increase in GFAP expression observed in the 273 μg V-treated group suggests that V exposure induces astrogliosis in this brain region. The elevated GFAP expression represents a key feature of neuroinflammation, reflecting astrocytic activation in response to toxic insults. Similar findings have been reported, with reactive gliosis observed in the hippocampus following V exposure, (Adekeye et al., 2020; García et al., 2005). The absence of significant differences between the control and lower concentration group in our study suggests that the exposure to lower doses might not be sufficient to trigger detectable astrocytic activation. Regarding MBP expression, although no significant differences were observed in the hippocampus in our study, previous research has reported evidence of myelin damage in this region associated with V exposure. V-induced neurotoxicity was shown to reduce MBP expression, leading to pale areas and discontinuities in myelin fibers (Adebiyi et al., 2019). This myelin disruption is often accompanied by axonal injury and glial cell activation, indicating a combined neuroinflammatory and neurotoxic response (Azeez et al., 2016). One possible explanation for the lack of significant MBP alterations in our study could be that the duration or dosage of V exposure in our experimental conditions was insufficient to induce demyelination. Additionally, myelin damage and its markers can exhibit regional heterogeneity, which might affect detectability in specific brain areas like the hippocampus (Azeez et al., 2016).

Studies on the effects of V exposure on oxidative stress in the hippocampus have revealed varied outcomes. Elevated LPO and increased ROS levels have been reported in sodium metavanadate-treated mice, accompanied by reductions in SOD and CAT activities, indicating compromised antioxidant defenses (García et al., 2004, 2005; Adebiyi et al., 2019). In contrast, Igado et al. (2012), observed increased activities of antioxidant enzymes (SOD, CAT, and GST) along with elevated GSH and LPO levels, suggesting an adaptive response to oxidative stress. In our study, however, no significant changes in ROS, LPO, or antioxidant enzyme activities (CAT, GPx, and SOD) were detected in the hippocampus. This divergence from previous findings may be explained by differences in exposure route, duration, and dosing. Moreover, under subchronic exposure conditions, cells within the hippocampus may engage compensatory mechanisms that help preserve redox homeostasis. Supporting this, although GSH and GSSG levels did not differ significantly, a slight increase was noted in the highest exposure group, along with a significant rise in GST activity, suggesting the activation of detoxification pathways even in the absence of classical oxidative stress markers. GST, in particular, is responsive to electrophilic and redox stress, and its upregulation may reflect an early or controlled oxidative response. This is consistent with reports indicating that mild or early oxidative stress may stimulate antioxidant defenses without causing measurable ROS accumulation or lipid peroxidation (He et al., 2022). Thus, our findings suggest that vanadium-induced redox stress in the hippocampus may have been effectively countered by endogenous protective systems, leading to an absence of evident oxidative imbalance. Still, the absence of significant alterations in classical oxidative stress markers is, in itself, a relevant finding. It suggests that vanadium exposure, under the conditions tested, does not markedly disrupt redox homeostasis. While our study focused on a selected panel of established oxidative stress indicators, future work employing broader and more sensitive approaches—such as transcriptomic or proteomic analyses—may help uncover more subtle or upstream compensatory responses.

### 4.4 Olfactory dysfunction and behavioral alterations

Several studies have shown that the V exposure can impair olfactory function. A variety of methods, such as tests for odor recognition memory, odor identification, and odor discrimination, have been applied to examine the extent of olfaction deficits resulting from V toxicity. In our study, we observed that rats exposed to both doses exhibited olfactory deficits, demonstrating an inability to detect a hidden food pellet in the cage bedding compared to the control group. Particularly, rats exposed to the higher concentration exceeded the test time limit, indicating more significant olfactory impairments than those observed in the lower-dose group. Although our study involved rats and employed a nose-to-brain delivery method, our findings are consistent with research by Ngwa et al. (2014) 2024, who administered 182 μg of V_2_O_5_ by nose drops with a similar protocol. They observed that V-treated male mice spent significantly less time interacting with female bedding, indicating marked olfactory impairment. Similarly, Colín-Barenque et al. (2015) 2018 demonstrated olfactory deficits following the administration of 0.02 M V_2_O_5_ through inhalation in an acrylic chamber for 1 h over 2 and 4 weeks. In the olfactory habituation/cross-habituation test, exposed rats failed to exhibit decreased durations of investigative behaviour upon repeated exposure to the same odor (habituation) and did not show increased investigative behavior when presented with a novel odor (cross-habituation), indicating diminished olfactory sensitivity. Furthermore, the burrowing test results suggest that intranasal V exposure significantly impairs natural burrowing behavior in rats. This behavior serves as a sensitive measure of hippocampal function, as lesions or dysfunction in this region are known to inhibit burrowing (Deacon, 2006). The significant reduction in burrowing activity observed in the high-concentration group highlights potential V-induced neurotoxicity affecting the hippocampus, although olfactory dysfunction may also contribute to the reduced burrowing activity. To further elucidate hippocampal involvement, future studies should include memory-based behavioral tasks to better assess hippocampal dysfunction following vanadium exposure.

The olfactory and behavioral deficits observed in our study align closely with the structural, histological, and biochemical findings. The significant loss of dopaminergic neurons in the OB, along with astrogliosis and disrupted myelin sheaths, underscores the direct impact of V exposure on olfactory processing. Similarly, histopathological changes in the OE support the link between peripheral nasal injury and impaired olfactory function. The increase of oxidative stress markers and upregulated GST activity, particularly at higher V concentrations, suggests a compensatory response to oxidative stress in the OB and hippocampus, critical regions for olfactory and cognitive functions. The reduction in burrowing behavior reinforces the neurotoxic effects of V exposure on hippocampal integrity. Together, these results indicate that the olfactory and behavioral impairments result from a combination of structural damage, oxidative stress, and neuroinflammatory processes affecting the olfactory and hippocampal regions.

Although the observed alterations in the OE are attributable to local exposure, previous studies have shown that systemically administered V can cross the BBB and accumulate in various brain regions, including the OB and hippocampus (Colín-Barenque et al., 2015, 2018; Folarin et al., 2017; Mustapha et al., 2023). Thus, effects in these structures may also reflect systemic distribution via paracellular transport. This dual potential for both direct epithelial toxicity and CNS accumulation complicates the interpretation of the observed olfactory deficits. Local epithelial damage may directly disrupt odor detection in the periphery. At the same time, V-induced effects on the OBs or other central structures may further contribute to the observed functional impairments.

In summary, this study provides evidence of V-induced olfactory and neurobehavioral changes using male rats as a model, allowing interpretation of the observed effects. Nevertheless, it is important to recognize that sex-related differences may influence olfactory function and susceptibility to environmental toxicants. Research in both humans and rodents indicates that females often exhibit enhanced olfactory sensitivity, and electrophysiological studies have revealed sex-dependent patterns in olfactory bulb activity (Maheshwar et al., 2025). These findings suggest functional sex differences. Therefore, while our model effectively demonstrates neurotoxic effects, future studies should include both sexes to better understand sex-specific responses and enhance translational relevance.

## 5 Conclusion

Our study provides a comprehensive characterization of vanadium-induced neurotoxicity in the olfactory system using a NTB delivery model in rats. Compared to traditional models that rely on systemic administration or inhalation exposure, our method enables the direct deposition of V on the OE, ensuring targeted exposure to the olfactory pathway. Although systemic absorption may still occur, this approach can be a valuable tool for studying region-specific effects. Inhalation models, while relevant for occupational and environmental exposures, often introduce variability in deposition efficiency, making dose standardization challenging. Systemic administration, on the other hand, results in widespread toxicant distribution, interfering with the isolation of olfactory-specific effects. The NTB method, however, offers a more controlled and reproducible approach to studying olfactory dysfunction, enabling the investigation of neurotoxicant-induced impairments with high anatomical specificity. This makes it a valuable tool for exploring the mechanisms underlying olfactory dysfunction and its potential links to neurodegenerative diseases. Using this approach, we assessed how different vanadium doses influence olfactory function and neurotoxicity. Our results demonstrated that the higher dose resulted in severe histological alterations, including extensive olfactory neuronal loss, significant thinning of the OE, significant loss of dopaminergic neurons, increased astrogliosis, disrupted myelin integrity in the OBs, and elevated oxidative stress markers. These changes were accompanied by marked olfactory deficits and impairments in hippocampus-dependent behaviors. Conversely, the lower dose induced milder but still significant olfactory impairments, characterized by moderate histopathological changes, and a more subtle oxidative response. The differential effects observed between the two doses suggest that each concentration may be useful for different types of studies. The higher dose could serve as a robust model for investigating severe neurotoxicity, OB degeneration, and extensive neuroinflammatory responses, making it suitable for studies focused on advanced olfactory dysfunction and its implications in neurodegenerative conditions. In contrast, the lower dose may be valuable for examining early-stage or mild olfactory impairments, which could be particularly relevant for understanding the initial effects of neurotoxicant exposure or for testing potential neuroprotective interventions. Importantly, despite the observed histological and functional impairments, the OE possesses intrinsic regenerative capacity. OSNs are continuously renewed under physiological conditions, and this ability may allow for partial recovery depending on the severity and duration of the insult. However, chronic inflammation or repeated exposure could compromise this regenerative process. Although a recovery phase was not included in this study, future research should aim to evaluate the reversibility of V-induced damage and the long-term regenerative dynamics of the OE.

In conclusion, our study establishes a novel NTB-based model for V-induced olfactory dysfunction, demonstrating dose-dependent neurotoxic effects that affect olfactory and cognitive regions. This model offers a valuable tool for advancing research into olfactory impairments, neuroinflammation, and oxidative stress, providing insights into potential mechanisms underlying olfactory dysfunction in both toxicological and neurodegenerative contexts. In future studies, we intend to apply this model in longitudinal experiments to better understand the progression of V-induced changes and evaluate their potential reversibility. Moreover, combining behavioral assessments with molecular and histopathological endpoints will allow for a more comprehensive understanding of the underlying mechanisms. Such integrative approaches will be essential for translating these findings into broader neuropathological contexts.

## Data Availability

The raw data supporting the conclusions of this article will be made available by the authors, without undue reservation.
